# Association and function analysis of genetic variants and the risk of gestational diabetes mellitus in a southern Chinese population

**DOI:** 10.3389/fendo.2024.1476222

**Published:** 2024-12-24

**Authors:** Qiulian Liang, Yan Sun, Ming Li, Ruiqi Li, Lijie Nie, Lin Lin, Xiangyuan Yu

**Affiliations:** ^1^ School of Public Health and Guangxi Key Laboratory of Diabetic Systems Medicine, Guilin Medical University, Guilin, China; ^2^ Department of Histology and Embryology, School of Basic Medicine, Hunan University of Medicine, Huaihua, China; ^3^ The Fujian Maternity and Child Health Hospital, College of Clinical Medicine for Obstetrics and Gynecology and Pediatrics, Fujian Medical University, Fuzhou, China

**Keywords:** gestational diabetes mellitus, genetic variants, association, nomogram model, prediction

## Abstract

**Background:**

Gestational diabetes mellitus (GDM) is a complex metabolic disease that has short-term and long-term adverse effects on mothers and infants. However, the specific pathogenic mechanism has not been elucidated.

**Objective:**

The aim of this study was to confirm the associations between candidate genetic variants (rs4134819, rs720918, rs2034410, rs11109509, and rs12524768) and GDM risk and prediction in a southern Chinese population.

**Methods:**

Candidate variants were genotyped in 538 GDM cases and 626 healthy controls. The odds ratio (OR) and its corresponding 95% confidence interval (CI) were calculated to assess the associations between genotypes and GDM risk. Then, the false-positive report probability (FPRP) analysis was adopted to confirm the significant associations, and bioinformatics tools were used to explore the potential biological function of studied variants. Finally, risk factors of genetic variants and clinical indicators identified by logistics regression were used to construct a nomogram model for GDM prediction.

**Results:**

It was shown that the *XAB2* gene rs4134819 was significantly associated with GDM susceptibility (CT vs. CC: adjusted OR = 1.38, 95% CI: 1.01–1.87, *p* = 0.044; CT/TT vs. CC: crude OR = 1.42, 95% CI: 1.08–1.86, *p* = 0.013). Functional analysis suggested that rs4134819 can alter the specific transcription factors (CPE bind and GATE-1) binding to the promoter of the *XAB2* gene, regulating the transcription of *XAB2*. The nomogram established with factors such as age, FPG, HbA1c, 1hPG, 2hPG, TG, and rs4134819 showed a good discriminated and calibrated ability with an area under the curve (AUC) = 0.931 and a Hosmer–Lemeshow test *p*-value > 0.05.

**Conclusion:**

The variant rs4134819 can significantly alter the susceptibility of the Chinese population to GDM possibly by regulating the transcription of functional genes. The nomogram prediction model constructed with genetic variants and clinical factors can help distinguish high-risk GDM individuals.

## 1 Introduction

Gestational diabetes mellitus (GDM) is defined as any degree of glucose intolerance with onset or first recognition during pregnancy (1). Studies have reported that GDM prevalence is approximately 14.0% globally and that it is approximately 14.8% in Mainland China (2, 3). GDM is considered to be associated with multiple adverse outcomes during pregnancy and childbirth in pregnant women. Hyperglycemic mothers are more likely to develop polyhydramnios, pre-eclampsia, obstructed labor, cesarean section, uterine prolapse, and infections, among others (4), while their offspring may be more prone to suffer from spontaneous abortion, stillbirth, congenital malformation, shoulder dystocia, birth injuries, infant respiratory distress syndrome, and macrosomia, to name a few (5). Moreover, GDM parturient and their children are both at a high risk of developing type 2 diabetes, obesity, metabolic syndrome, and cardiovascular diseases in later life (6). GDM poses a serious threat to maternal and infant health, but its etiology is still not fully understood.

Considering the adverse effects of GDM on mothers and fetuses, it is important to develop reasonable strategies to identify and intervene high-risk individuals to reduce the incidence rate of GDM. Currently, the well-established risk factors of GDM can be advanced maternal age, pre-pregnancy overweight or obesity, family history of T2DM, history of GDM, parity, polycystic ovary syndrome (PCOS), ethnicity, diet, and physical activity, among others (7). Among these risk factors, heredity plays an indispensable role. A study conducted in southern China reported that the GDM risk of pregnant women with a family history of diabetes in first-degree relatives were at 2.52 times higher than those without the history (8). Furthermore, Wan et al. showed that Chinese women migrating to Australia had an elevated risk at developing GDM compared to Australian-born Caucasian women (9). In addition, Asian women had a higher risk of GDM than Caucasian women. This further emphasized the importance of genetic background in the pathogenesis of GDM (10).

Genetic studies such as candidate gene studies and genome-wide association studies (GWASs) have constantly identified the DNA sequence variant [single-nucleotide polymorphism (SNP)], which might play a role in altering the promoter and enhancer activity, alternative splicing, mRNA conformation and its posttranscription level, protein function, etc., leading to individual differences in disease susceptibility (11–13). To date, studies including newly two large-scale GWASs performed in east Asia and Finland have detected numerous GDM-associated SNPs (14, 15), for instance, *MTNR1B* gene rs10830963, *CDKAL1* rs7766070, *TCF7L2* rs34872471, *CDKN2B* rs1333051, *CMIP* rs2926003, and *CPO* rs1597916. In preliminary studies, we also have identified a series of GDM genetic polymorphisms in the Guilin population, such as the *OR2D2* gene rs1965211, *RXR-γ* rs2134095, *TSNARE1* rs7814359, *XAB2* rs3760675, *ERBB4* rs1595066, *MTNR1B* rs10830963, *CDKAL1* (rs7756992 and rs7754840), and *ACE2* (rs6632677 and rs2074192) (16–21). These variants were considered to significantly affect individuals′ susceptibility to GDM by influencing gene expression or interacting with age, pre-pregnancy BMI, blood glucose, or lipid levels.

The clinical practice of GDM screening and diagnosis focuses on 24–28 weeks, which is already in the middle and late stages of pregnancy and cannot prevent the pathological and physiological processes of GDM (22). Thus, a rational strategy of GDM prevention in early pregnancy was desired for clinical application. A nomogram model is a method that can predict the probability of disease outcome events that may occur in individuals with specific characteristics in the future (23). Previously predictive models of GDM were constructed based on the maternal demographic and clinical indicators during early pregnancy, such as age, pre-pregnancy BMI, parity, FPG, and other blood test indicators (24–26). Even though the performances of their model were acceptable, these studies did not comprehensively consider genetic background of pregnant women in the model. Therefore, a risk predictive model containing both genetic and environment components was essential to improve clinicians’ decision for individualized early prevention and intervention of GDM.

This case–control study aimed to detect the associations between selected functional variants and clinical traits and GDM risk. Then, a nomogram prediction model based on the GDM positively associated genetic and clinical markers was constructed, and its diagnostic efficacy was evaluated.

## 2 Materials and methods

### 2.1 Subjects

**Figure 1:**
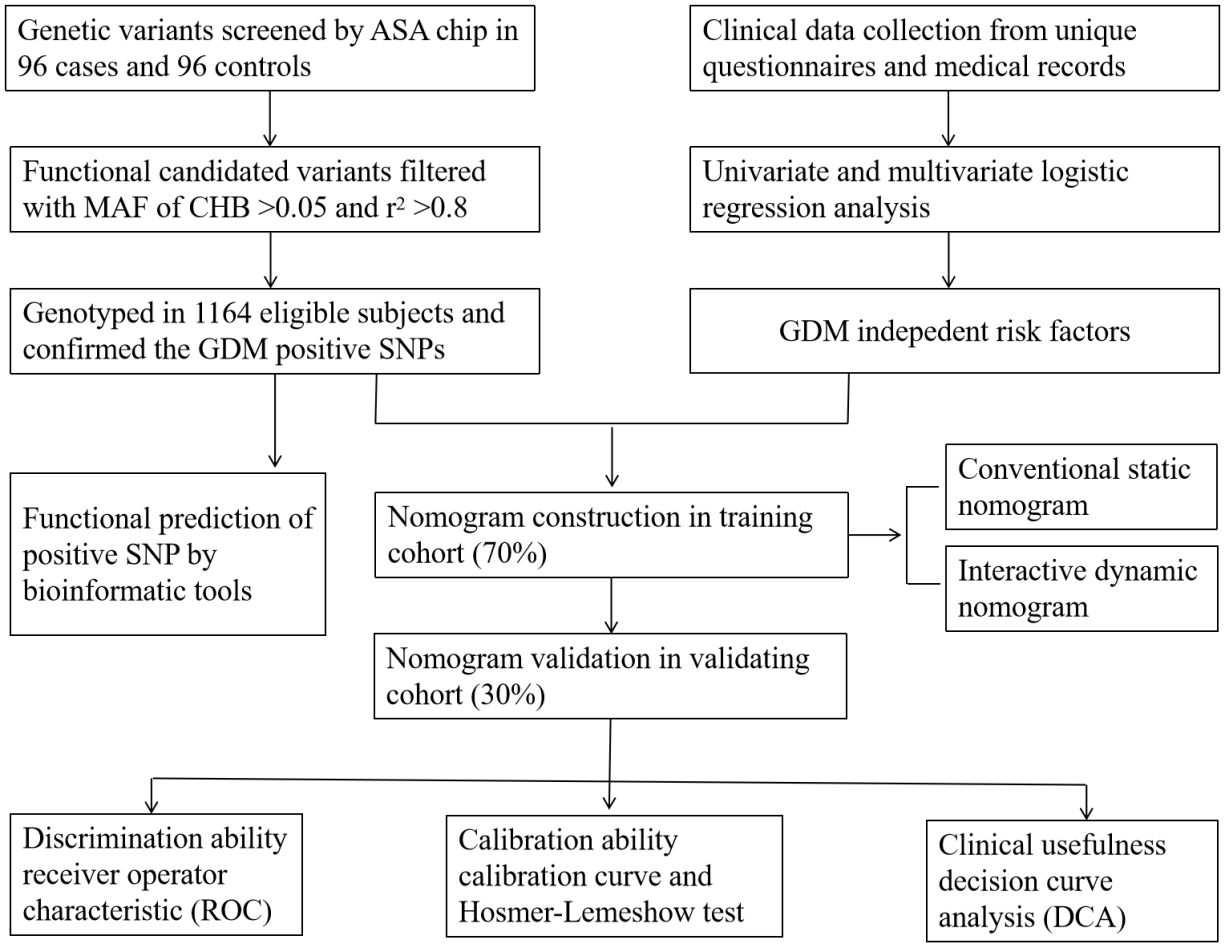
Design process of the study. ASA chip, infinium Asian Screening Array (ASA, illumina) BeadChip; MAF, minimum allele frequency. CHB, the Chinese Han population in Beijing. *r* ^2^ was the index of linkage disequilibrium.

A total of 1,164 participants in early pregnancy, namely, 538 GDM cases and 626 healthy controls, aged 18–45 years, were recruited in the Affiliated Hospital of Guilin Medical University between September 2014 and April 2016. A standard 75-g oral glucose tolerance test (OGTT) was conducted at 24–28 weeks of gestation, and according to the criteria recommended by the International Association of Diabetes and Pregnancy Study Groups (IADPSG), GDM was diagnosed if any of the three threshold values was reached or exceeded: fasting plasma glucose (FPG) ≥5.1 mmol/L, 1-hour plasma glucose (1hPG) ≥10.0 mmol/L, and 2-hour plasma glucose (2hPG) ≥8.5 mmol/L (27). Moreover, subjects in our study should satisfy the following requirements: singleton pregnancy, local residents, and having no kinship with each other. Pregnant women who were progestationally diagnosed as having endocrine and metabolic diseases such as type 1 or type 2 diabetes, and have used long-term glucose metabolism-affecting drugs before pregnancy were excluded. The present study was approved by the Ethics Committee of Guilin Medical University (number GLMC20131205) and conducted according to the principles of the Declaration of Helsinki. The study design is shown in **Figure 1**.

### 2.2 Data collection

Participants′ information such as age, pre-pregnancy weight, height, systolic blood pressure (SBP), diastolic blood pressure (DBP), hemoglobin A1c (HbA1c), blood glucose levels (FPG, 1hPG, and 2hPG) and triglyceride (TG), total cholesterol (TC), low-density lipoprotein cholesterol (LDL-c), and high-density lipoprotein cholesterol (HDL-c) levels were collected from structured questionnaires and hospital medical records. Pre-pregnancy body mass index (BMI) was calculated as pre-pregnancy weight (kg) divided by the square of height (m).

### 2.3 Genomic DNA extraction

The genomic DNA was extracted from EDTA-treated peripheral whole blood using the Aidlab DNA extraction kit (Aidlab Biotechnologies Co., Ltd, China) and stored at −80°C before polymerase chain reaction (PCR).

### 2.4 Candidate variants selection and genotyping

**Figure 2:**
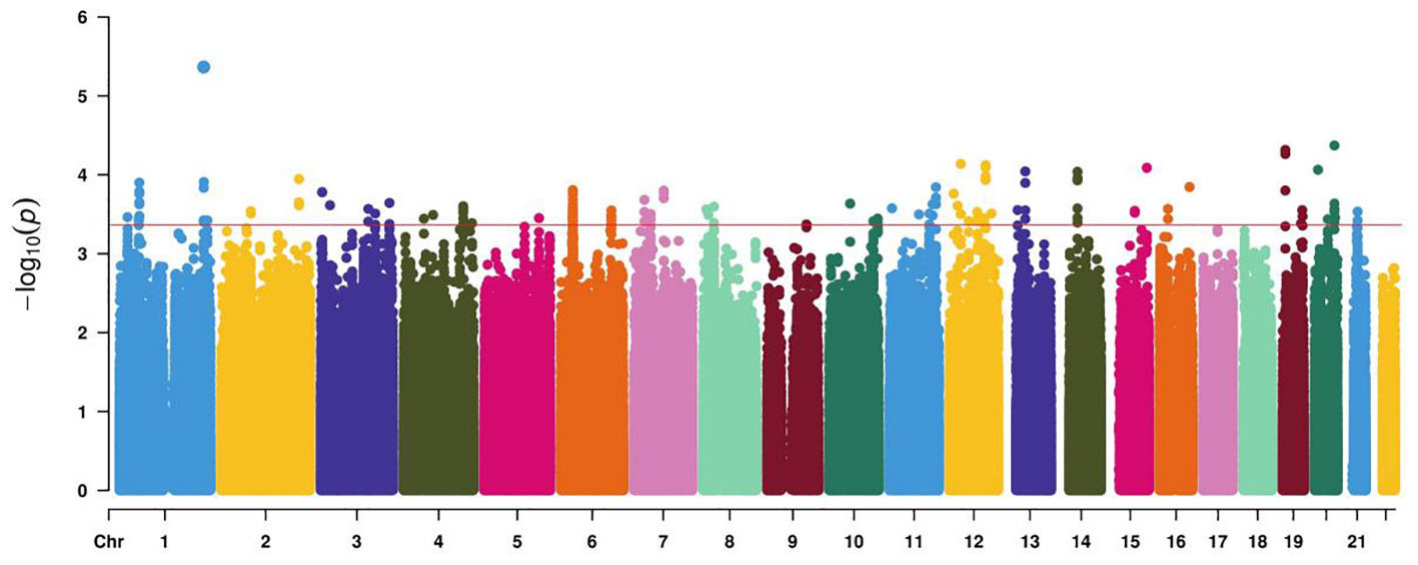
Manhattan plot demonstrating the −log10 *p*-value for GDM-associated SNPs at the discovery stage. The red line represents a genome-wide significance threshold (*p* = 5×10^−4^).

After conducting Infinium Asian Screening Array (ASA, Illumina) BeadChip analysis on 96 cases of GDM and controls, a series of genetic variants were detected at the test level of 10^−4^ (**Figure 2**). Candidate genetic variants must meet the following conditions: located in the current functional region of the genome, such as transcription factor binding sites (TFBS), splicing sites (SS), and miRNA binding sites (MBS); the minimum allele frequency (MAF) in the Chinese Han population in Beijing (CHB) is greater than 5%; and the linkage disequilibrium (LD) coefficient *r* ^2^ between variants is less than 0.8. If *r* ^2^ > 0.8, only TagSNP is selected. Finally, five functional polymorphisms were selected, of which four (rs4134819, rs720918, rs11109509, and rs12524768) were located at TFBS, and one (rs2034410) was located at miRNA binding sites (**Supplementary Table S1**).

Candidate variants were genotyped by the Sequenom MassARRAY Platform. The PCR master mix was composed of 1 μL of template DNA (20–100 ng/μL), 1.850 μL of ddH_2_O, 0.625 μL of 1.25×PCR buffer (15 mmol/L MgCl_2_), 0.325 μL of 25 mmol/L MgCl_2_, 0.1 μL of 25 mmol/L dNTP mix, 1 μL of 0.5 mmol/L primer mix, and 0.1 μL of 5 U/mL HotStar Taq polymerase. The reaction was conducted at 94°C for 15 min, followed by 45 cycles at 94°C for 20 s, 56°C for 30 s, and 72°C for 1 min, with a final incubation at 72°C for 3 min. The PCR primers are listed in **Supplementary Table S2**.

### 2.5 Functional analysis

As predicted by the SNPinfo Web Server (https://manticore.niehs.nih.gov/snpinfo/snpfunc.html) (28), rs4134819 was located at TFBS. We thus used the Alibaba 2.1 tools (http://gene-regulation.com/pub/programs/alibaba2/index.html) to predict the potential functional influence (29). In addition, expression quantitative trait loci (eQTL) analysis was adopted to observe the effect of rs4134819 on the expression regulation of *XAB2* gene using VarNote-REG (http://www.mulinlab.org/varnote/application.html#REG) (30) online tools.

### 2.6 Statistical analysis

Data were processed using the IBM SPSS Statistics 28 for Windows (IBM Corp., Armonk, NY, USA) and R software (4.3.1). Continuous variables according to normal distribution were described as mean ± standard deviation (mean ± SD) and independent samples Student’ s *t*-test was used to compare the difference between case and control groups, while variables with non-normal distribution were presented as median (interquartile range) and Mann–Whitney *U* test was utilized for difference comparison between the two groups. The chi-square (χ^2^) test was used for categorical variables. The Hardy–Weinberg Equilibrium (HWE) assessed by the χ^2^ goodness of fit was conducted to determine whether the genotype frequencies are in equilibrium in the control group. Odds ratios (ORs) and their corresponding 95% confidence intervals (CIs) were employed to evaluate the associations between variants and GDM risk. Stratified analysis was carried out to detect the relationship between positive SNP and GDM risk in specific subgroups based on the mean value of clinical variables. A two-tailed test with *p* < 0.05 indicates that the difference is statistically significant. The false-positive report probability (FPRP) analysis was also performed to assess the significant associations. A cutoff value of 0.2 and a prior probability level of 0.1 were preset to observe an OR of 1.5 for the combined genotypes with an increased risk. Only the *p*_FPRP_ < 0.2 can be considered as genuine association (31).

Clinical variables with a *p* < 0.05 in univariate logistics regression can be subsequently incorporated in multifactorial regression analysis to further detect the GDM risk factors. The subjects were randomly split into two groups (training cohort and validating cohort) at a ratio of 7:3, and the nomogram model was constructed to predict GDM occurrence using the “rms” package in the training cohort. The receiver operator characteristic (ROC) curve and the area under the curve (AUC) were produced to evaluate the predictive performance of the nomogram. The calibration curve conducted via a bootstrap method with 1,000 resamples and the Hosmer–Lemeshow test were used to assess the level of consistency between the predicted probabilities and the observed outcomes. Furthermore, the clinical validity and net benefit of the nomogram were appraised by adopting a decision curve analysis (DCA). To facilitate the clinical implementation and application of the risk model, we developed an interactive dynamic nomogram based on the “DynNom” package and a web application “shinyapps” (https://www.shinyapps.io/).

## 3 Results

### 3.1 Subjects’ characteristics

The anthropometric and biochemical materials were significantly different between case and control groups. Compared to the control group, age, pre-BMI, blood pressure levels (SBP and DBP), blood glucose, and lipid metabolism levels (FPG, 1hPG, 2hPG, HDL-c, LDL-c, and TG) were higher in the case group (*p* < 0.05), as shown in **Table 1**.

**Table 1:** The baseline characteristics of subjects (mean ± SD).

Clinical variables	GDM (*n* = 538)	Control (*n* = 626)	*t*	*p*
Age (years old)	31.46 ± 4.74	28.82 ± 4.14	10.05	<0.001
Pre-BMI (kg/m^2^)	23.13 ± 3.62	21.44 ± 3.01	8.53	<0.001
SBP (mmHg)	111.52 ± 10.59	108.75 ± 9.39	4.67	<0.001
DBP (mmHg)	70.35 ± 8.70	68.66 ± 7.94	3.45	0.001
FPG (mmol/L)	5.22 ± 1.33	4.41 ± 0.37	13.65	<0.001
1hPG (mmol/L)	9.74 ± 2.25	6.95 ± 1.43	24.83	<0.001
2hPG (mmol/L)	8.28 ± 2.16	6.07 ± 1.10	21.46	<0.001
HbA1c (%)	5.43 ± 0.68	5.00 ± 0.49	12.26	<0.001
TG (mmol/L)	2.66 ± 1.20	2.42 ± 1.00	3.71	<0.001
TC (mmol/L)	5.36 ± 1.16	5.30 ± 1.08	0.91	0.361
HDL-c (mmol/L)	1.66 ± 0.42	1.65 ± 0.40	0.17	0.863
LDL-c (mmol/L)	3.48 ± 1.02	3.46 ± 1.01	0.29	0.773

Pre-BMI, pre-pregnancy body mass index; SBP, systolic blood pressure; DBP, diastolic blood pressure; FPG, fasting plasma glucose; TG, triglyceride; TC, total cholesterol; HDL-c, high-density lipoprotein cholesterol; LDL-c, low-density lipoprotein cholesterol.

### 3.2 Association between variants and GDM risk

Genotype frequencies of variants (rs4134819, rs720918, rs11109509, and rs12524768) followed the principle of HWE (*p*_HWE_ > 0.05) except for rs2034410 (**Table 2**). Only the genotype distribution of rs4134819 was different between GDM cases and healthy controls (χ^2^ = 6.83, *p* = 0.033). After adjusting age and pre-BMI, the rs4134819 CT genotype could significantly increase GDM risk compared to the CC genotype (adjusted OR = 1.38, 95% CI: 1.01–1.87, *p* = 0.044). Under the dominant model, CT/TT genotypes could increase the GDM risk by 42% compared with the CC genotype (crude OR = 1.42, 95% CI: 1.08–1.86, *p* = 0.013). However, after adjusting for age and pre-BMI, this significant association disappeared (**Table 2**).

**Table 2:** The associated analysis of GDM and screened functional variants.

Genotype	GDM	Control	*p*_HEW_	*p ^a^*	Crude OR (95% CI)	*p ^b^*	Adjusted OR (95% CI)	*p ^c^*
rs4134819								
CC	114	169	0.107	0.033	1		1	
CT	273	275			1.47 (1.10–1.97)	**0.009**	1.38 (1.01–1.87)	**0.044**
TT	129	146			1.31 (0.94–1.83)	0.115	1.26 (0.88–1.81)	0.199
CT/TT	402	421		0.013	1.42 (1.08–1.86)	**0.013**	1.34 (0.99–1.79)	0.051
CC/CT	387	444		0.922	1		1	
TT	129	146			1.01 (0.77–1.33)	0.92	1.03 (0.76–1.37)	0.871
rs720918								
AA	310	353	0.794	0.943	1		1	
AG	196	232			0.96 (0.75–1.23)	0.756	0.94 (0.73–1.22)	0.658
GG	30	36			0.95 (0.57–1.58)	0.840	1.02 (0.59–1.74)	0.955
AG/GG	226	268		0.734	0.96 (0.76–1.21)	0.734	0.95 (0.74–1.22)	0.701
AA/AG	506	585		0.884	1		1	
GG	30	36			0.96 (0.59–1.59)	0.884	1.04 (0.61–1.76)	0.886
rs2034410								
TT	410	474	<0.05	0.649	1		1	
TC	68	80			0.98 (0.69–1.39)	0.922	1.09 (0.75–1.59)	0.659
CC	26	23			1.31 (0.73–2.33)	0.363	1.50 (0.82–2.76)	0.190
TC/CC	94	103		0.734	1.06 (0.77–1.44)	0.734	1.18 (0.85–1.65)	0.323
TT/TC	478	554		0.355	1		1	
CC	26	23			1.31 (0.74–2.33)	0.356	1.48 (0.81–2.72)	0.202
rs11109509								
AA	264	298	0.377	0.357	1		1	
AG	193	248			0.88 (0.68–1.13)	0.311	0.88 (0.67–1.15)	0.334
GG	62	61			1.15 (0.78–1.70)	0.490	1.11 (0.73–1.68)	0.622
AG/GG	255	309		0.553	0.93 (0.73–1.18)	0.553	0.92 (0.72–1.19)	0.533
AA/AG	457	546		0.309	1		1	
GG	62	61			1.21 (0.84–1.77)	0.310	1.18 (0.79–1.76)	0.426
rs12524768								
GG	409	471	0.376	0.192	1		1	
GA	114	146			0.90 (0.68–1.19)	0.455	0.90 (0.67–1.21)	0.472
AA	14	8			2.02 (0.84–4.85)	0.118	2.02 (0.79–5.20)	0.143
GA/AA	128	154		0.750	0.96 (0.73–1.25)	0.750	0.95 (0.72–1.27)	0.749
GG/GA	523	617		0.098	1		1	
AA	14	8			2.07 (0.86–4.96)	0.105	2.07 (0.81–5.32)	0.129

ASA chip, infinium Asian Screening Array (ASA, illumina) BeadChip; HWE, Hardy–Weinberg Equilibrium test; a, Genotype distribution difference tested by χ^2^; b, Unconditional logistic regression analysis; c, Adjusted for age, pre-BMI in logistics regression models. Bold values indicate that the differences are statistically significant.

The stratified analysis was performed under the dominant model. Compared to the CC genotype, CT/TT genotypes had a higher GDM risk in subgroups with SBP > 110.03 mmHg (adjusted OR = 1.81, 95% CI: 1.18–2.78, *p* = 0.007), DBP > 69.44 mmHg (adjusted OR = 1.60, 95% CI: 1.03–2.47, *p* = 0.036), 2hPG > 7.10 mmol/L (adjusted OR =1.85, 95% CI:1.12–3.04, *p* = 0.016), HbA1c > 5.20% (adjusted OR = 1.65, 95% CI: 1.03–2.64, *p* = 0.036), and TG > 2.53 mmol/L (adjusted OR = 1.59, 95% CI: 1.03–2.46, *p* = 0.038) after adjusting age and pre-BMI (**Table 3**).

**Table 3:** Stratified analysis for associations between *XAB2* gene rs4134819 and GDM risk.

Variables	CC (case/control)	CT/TT (case/control)	Crude OR (95%CI)	*p* ^a^	Adjusted OR (95% CI)	*p* ^b^
Age (years old)						
≤30.04	53/122	177/290	1.41 (0.97–2.04)	0.074	1.44 (0.97–2.12)	0.069
>30.04	61/46	225/130	1.31 (0.84–2.03)	0.235	1.21 (0.78–1.90)	0.397
Pre-BMI (kg/m^2^)						
≤22.22	59/119	168/279	1.22 (0.84–1.75)	0.298	1.24 (0.85–1.82)	0.272
>22.22	55/49	234/141	1.48 (0.95–2.29)	0.080	1.44 (0.92–2.27)	0.111
SBP (mmHg)						
≤110.03	60/90	187/256	1.10 (0.75–1.60)	0.635	1.02 (0.68–1.52)	0.939
>110.03	54/78	215/164	1.89 (1.27–2.83)	**0.002**	1.81 (1.18–2.78)	**0.007**
DBP (mmHg)						
≤69.44	62/93	194/229	1.27 (0.87–1.85)	0.209	1.18 (0.80–1.75)	0.409
>69.44	52/75	208/191	1.57 (1.05–2.35)	**0.029**	1.60 (1.03–2.47)	**0.036**
FPG (mmol/L)						
≤4.78	48/146	154/368	1.27 (0.87–1.86)	0.209	1.19 (0.80–1.76)	0.393
>4.78	66/22	248/52	1.59 (0.90–2.80)	0.109	1.65 (0.92–2.96)	0.096
1hPG (mmol/L)						
≤8.24	23/133	95/335	1.64 (0.99–2.70)	0.051	1.55 (0.93–2.57)	0.091
>8.24	91/35	307/85	1.39 (0.88–2.20)	0.159	1.37 (0.85–2.19)	0.193
2hPG (mmol/L)						
≤7.10	32/135	106/357	1.25 (0.81–1.95)	0.318	1.17 (0.74–1.85)	0.502
>7.10	82/33	296/63	1.89 (1.16–3.08)	**0.010**	1.85 (1.12–3.04)	**0.016**
HbA1c (%)						
≤5.20	47/126	141/317	1.19 (0.81–1.76)	0.376	1.10 (0.74–1.65)	0.628
>5.20	67/42	261/103	1.59 (1.02–2.49)	**0.043**	1.65 (1.03–2.64)	**0.036**
TG (mmol/L)						
≤2.53	61/99	207/265	1.27 (0.88–1.83)	0.205	1.18 (0.80–1.75)	0.407
>2.53	53/69	195/155	1.64 (1.08–2.48)	**0.020**	1.59 (1.03–2.46)	**0.038**

The stratified analysis was conducted based on the mean value level of the variables. a, Unconditional logistic regression analysis; b, Adjusted for age, pre-BMI in logistics regression models. Bold values indicate that the differences are statistically significant.

However, there was no significant association observed between other variants (rs720918, rs2034410, rs11109509, and rs12524768) and the risk of GDM, as shown in **Table 2**.

### 3.3 FPRP analysis

The FPRP test was adopted to evaluate the robustness of positive associations with a prior probability setting at 0.1 and an FPRP threshold value setting at 0.2. As demonstrated in **Table 4**, the association between rs4134819 and GDM risk in the dominant model (CT/TT vs. CC) seems to be reliable correlation (*p* = 0.147), while other statistically significant results may be detected by chance and should be taken with caution.

**Table 4:** FPRP analysis for the positive associations between rs4134819 and GDM risk.

Comparison	Crude OR (95% CI)	Adjusted OR (95% CI)	Prior probability					
			0.25	0.1	0.01	0.001	0.0001	0.00001
CT vs. CC		1.38 (1.01–1.87)	0.154	0.353	0.857	0.984	0.998	1.000
CT/TT vs.CC	1.42 (1.08–1.86)		0.054	**0.147**	0.655	0.950	0.995	0.999
Subgroup								
SBP > 110.03 mmHg		1.81 (1.18–2.78)	0.095	0.239	0.776	0.972	0.997	1.000
DBP > 69.44 mmHg		1.60 (1.03–2.47)	0.217	0.454	0.902	0.989	0.999	1.000
2hPG > 7.10 mmol/L		1.85 (1.12–3.04)	0.187	0.409	0.884	0.987	0.999	1.000
HbA1c > 5.20 (%)		1.65 (1.03–2.64)	0.240	0.486	0.912	0.991	0.999	1.000
TG > 2.53 mmol/L		1.59 (1.03–2.46)	0.222	0.461	0.904	0.990	0.999	1.000

The prior probability of FPRP analysis is set to 0.1, and the statistical cutoff value is 0.2. Bold values indicate that the differences are statistically significant.

### 3.4 Biological functional analysis

**Figure 3:**
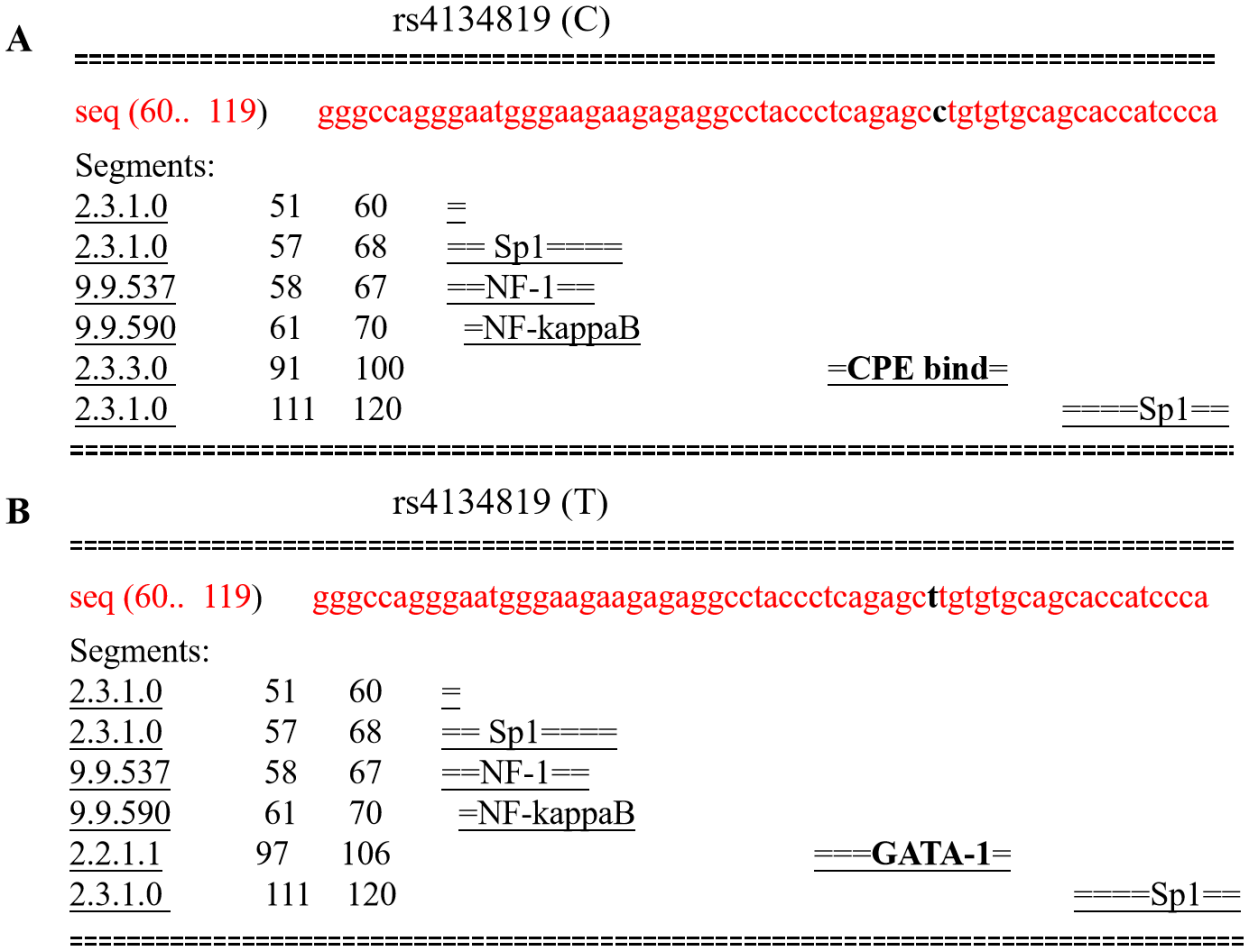
Comparison of transcription factors bound to rs4134819 C>T. **(A)** The transcription factors bound to the wild allele of rs4134819 in the 60–119 sequence. **(B)** As the wild allele (C) altered to rs4134819 T, the transcription factor changed from “CPE bind” to “GATA-1” in the 97–106 sequence.

Given the fact that rs4134819 is located at the TFBS region of *XAB2* gene, bioinformatic tools were used to predict the potential functional impact caused by the genetic variant. It can be seen that the rs4134819 C allele binds with the transcription factor “CPE bind” in 91–100 bp. However, when the wild-type allele changes to T allele, the transcription factor attached to it also changes to “GATA-1”, which suggested that rs4134819 may have an impact on the regulation of gene transcription (**Figures 3A, B**). Moreover, eQTL analysis based on the Genotype-Tissue Expression Project (GTEx V8) indicated that rs4134819 can be an eQTL and affect the expression level of functional genes such as *PET100*, *PCP2*, and *CTD-3214H19.6* in different tissues (**Table 5**).

**Table 5:** The eQTL effect analysis of rs4134819 regulating functional gene expression using online tools.

Position (rs4134819)	Gene name	Tissues	*p*
chr19:7628345	*PET100*	Whole blood	4.66×10^−16^
chr19:7628345	*PCP2*	Esophagus mucosa	3.66 ×10^−7^
chr19:7628345		Spleen	2.77×10^−6^
chr19:7628345		Skin (lower leg)	3.14×10^−6^
chr19:7628345	*CTD-3214H19.6*	Pancreas	4.68×10^−6^
chr19:7628345		Esophagus mucosa	3.98×10^−5^
chr19:7628345		Skin (lower leg)	6.04×10^−6^
chr19:7628345		Skin (suprapubic)	2.97×10^−7^

eQTL, expression quantitative trait loci.

### 3.5 Factor selection and the nomogram establishment

Clinical variable selection was based on univariate and multivariate logistic regression analysis. The latter was performed by a backward stepwise selection with the Akaike information criterion (AIC). The final results showed that age, FPG, HbA1c, 1hPG, 2hPG, and TG were independent risk factors of GDM with a model AIC of 536.78. Furthermore, considering that rs4134819 was significantly associated with GDM, we attempted to take the variant into the model. Surprisingly, the AIC of the multivariable logistic regression model was decreased to 533.94 (**Table 6**).

**Table 6:** GDM risk factors screened using univariate and multivariate logistic regression analysis.

Variables	Univariate analysis			Multivariate analysis		
	OR	95% CI	*p*	OR	95% CI	*p*
Age	1.15	1.11–1.19	<0.001	1.09	1.04-1.15	<0.001
Pre-BMI	1.17	1.11–1.22	<0.001	–	–	–
DBP	1.02	1.01–1.04	0.008	–	–	–
SBP	1.03	1.01–1.04	<0.001	–	–	–
FPG	11.68	8.16–16.72	<0.001	17.3	10.27–29.14	<0.001
1hPG	11.78	8.39–16.53	<0.001	4.33	2.63–7.12	<0.001
2hPG	14.19	9.99–20.16	<0.001	10.10	5.91–17.25	<0.001
HbA1c	5.46	3.70–8.07	<0.001	1.63	1.06–2.51	0.026
TG	1.30	1.12–1.50	<0.001	1.28	1.03–1.59	0.026
rs4134819DM	1.46	1.05–2.04	0.025	1.76	1.06–2.91	0.029

DM, dominant genetic model.

**Figure 4:**
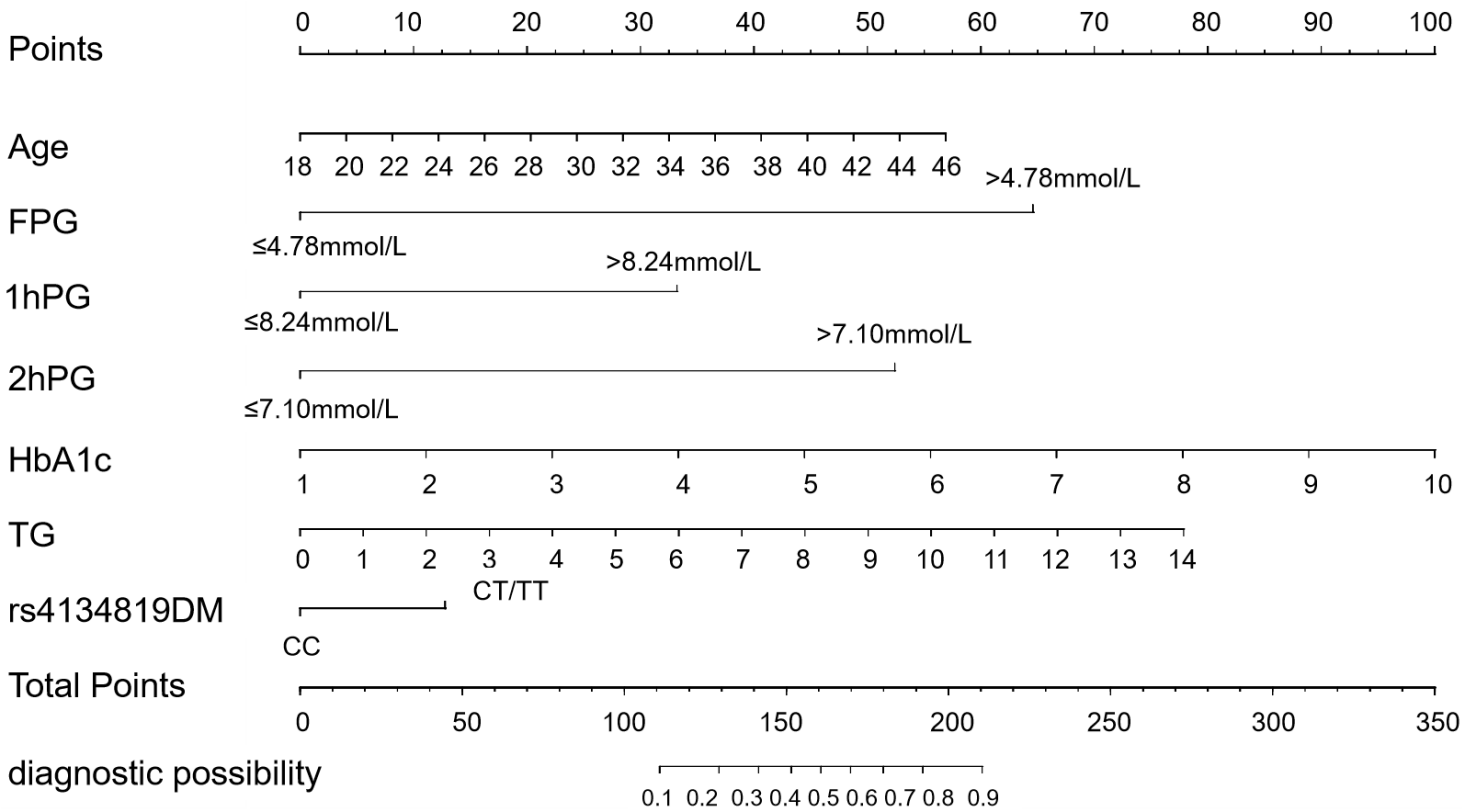
Conventional static nomogram model constructed with age, FPG, 1hPG, 2hPG, HbA1c, TG, and rs4134819DM (dominant model). A standard of scoring based on the regression coefficient (β) of indicators is formulated. Each level of the indicators will be given a specific score, and the scores of each factor are added up to get the total point. The value corresponding to the vertical line of the total points is the probability of GDM occurrence.

**Figure 5:**
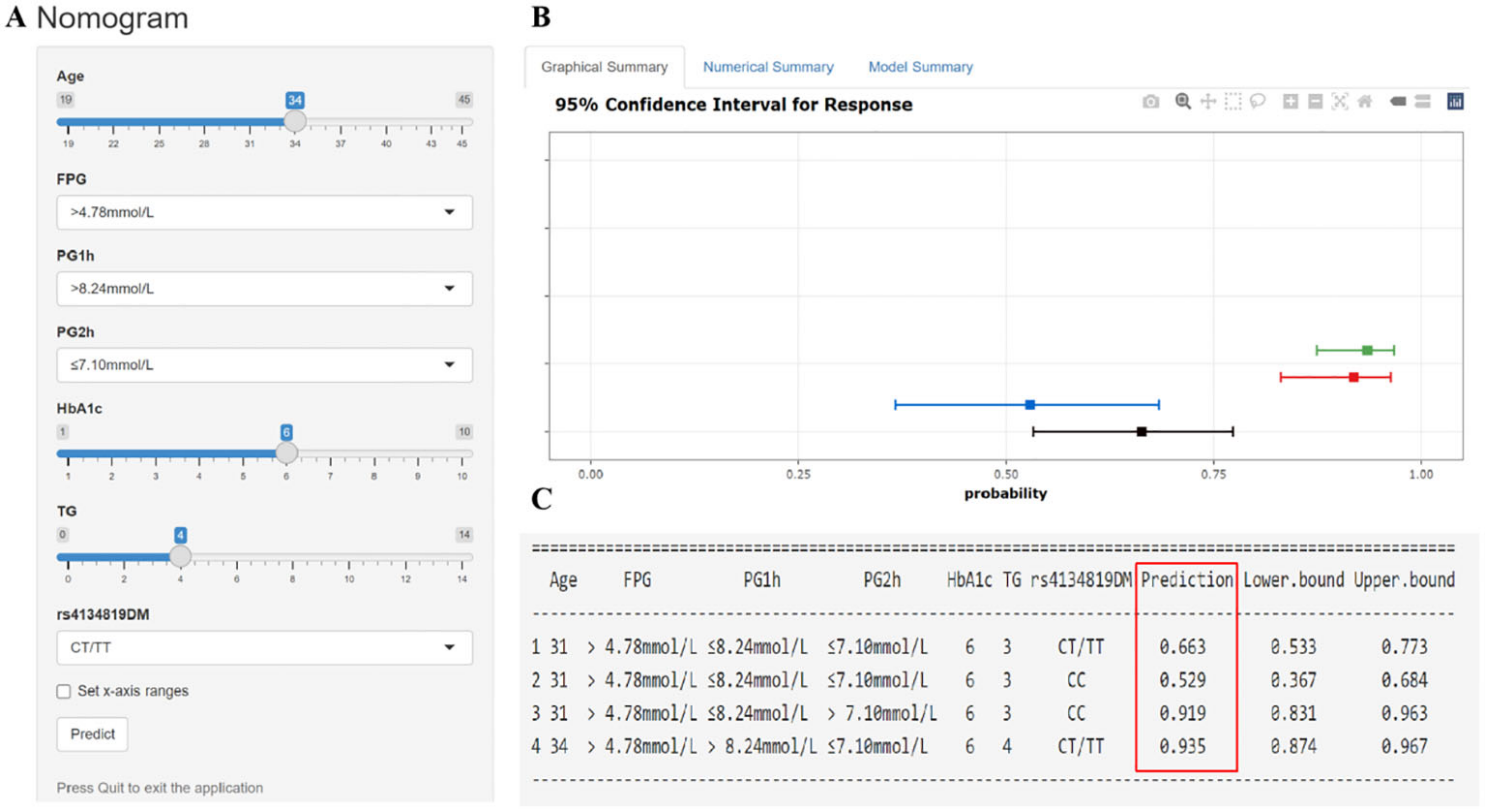
Interactive dynamic nomogram based on a web application “shinyapps” (https://qiulianl.shinyapps.io/GDM_risk_prediction/). **(A)** The value input plate of pregnant women’s predictive indicators. **(B)** The results of model prediction are presented in a visual form. A horizontal line represents the predictive results of one subject, the bold square dot is the probability of GDM, and the two ends of the line are 95% confidence intervals. **(C)** Presentation of specific input values and corresponding predictive result values (GDM incidence and 95% confidence interval).

The seven GDM risk factors were ultimately used to construct the predictive nomogram in the training cohort. In the traditionally static nomogram, each value level of the risk factors was given the corresponding score, and the total score obtained by adding up the score of all risk factors can be employed to predict the probability of GDM occurrence (**Figure 4**). Meanwhile, to make the nomogram more applicable and convenient for clinicians, we developed an online dynamic nomogram (https://qiulianl.shinyapps.io/GDM_risk_prediction/), which was able to visualize the GDM predictive results (**Figures 5A, B**). For instance, a 31-year-old pregnant woman had the following test results: FPG > 4.78 mmol/L, 1hPG > 8.24 mmol/L, 2hPG ≤ 7.10 mmol/L, HbA1c = 6%, and TG = 3 mmol/L, and carried CT/TT genotypes, whose probability of GDM occurrence was predicted as 66.3%. Interestingly, when the exposure level of clinical indicators remained unchanged and only the genotype was altered to CC, the predictive probability of GDM was 52.9%, which suggested that genetic component played a certain role in GDM occurrence (**Figure 5C**).

### 3.6 Validation of the nomogram

**Figure 6:**
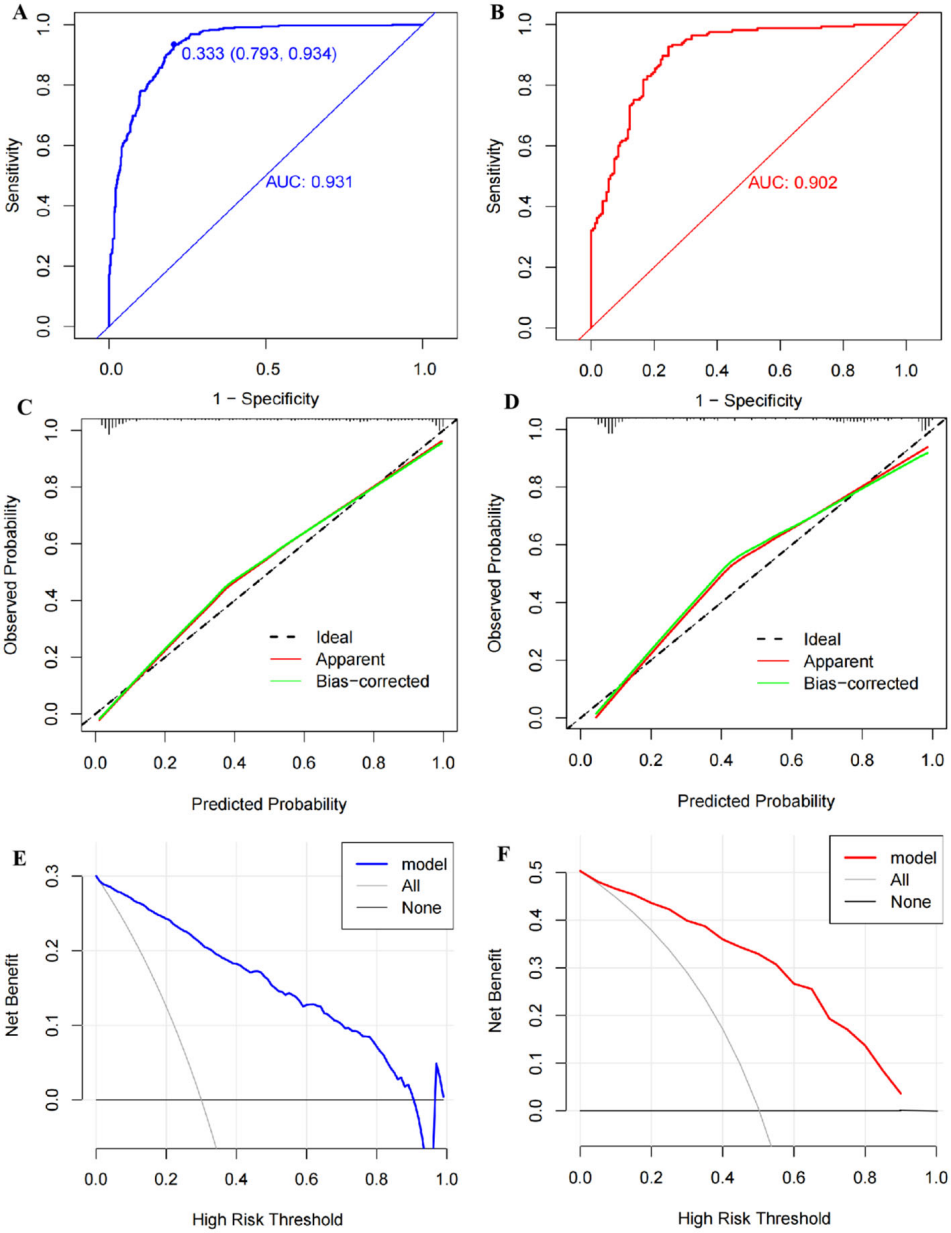
Validation of the nomogram model. **(A)** The receiver operator characteristic (ROC) curve with an area under the curve (AUC) of 0.931, a cutoff value of 0.333, a sensitivity of 0.934, and a specificity of 0.793 in the training set. **(B)** The ROC curve with an AUC of 0.902 in the testing set. **(C)** The calibration curve conducted via a bootstrap method with 1,000 resamples in the training set, and its mean absolute error was 0.027. **(D)** The calibration curve conducted by a bootstrap method with 1,000 resamples in the testing set, and the mean absolute error was 0.035. **(E)** Decision curve analysis (DCA) shows the clinical validity of the nomogram model in the training set. **(F)** DCA curve in the testing set.

The AUC of the nomogram was 0.931 (95% CI: 0.914–0.948) in the training cohort and 0.902 (95% CI: 0.870–0.935) in the internal validation cohort, suggesting a good predictive power of the model (**Figures 6A, B**). The calibration curves of the nomogram were close to the ideal line whether in the training cohort or the validation cohort, and the Hosmer–Lemeshow analysis also produced acceptable results (*p* > 0.05), indicating that there was a good calibration for the risk estimation in the predictive model (**Figures 6C, D**). The nomogram DCA curves were higher than the “treat all” and “treat none” lines for most of the predicted threshold probabilities, which showed good clinical validity and net benefit (**Figures 6E, F**).

## 4 Discussion

As is known, chronic insulin resistance and pancreatic β-cell dysfunction are the main physiopathological mechanisms of GDM. As pregnancy progresses, the upregulation of human placental lactogen (hPL), estrogen, progesterone, cortisol, and prolactin can cause progressive insulin resistance, and when pancreatic β-cells fail to compensate for the situation, it will lead to hyperglycemia and even gestational diabetes (32, 33). GDM is affected by the interplay between multiple etiologies and has an obvious genetic tendency (34). Individual genetic susceptibility often interacts with environment factors to participate in the onset of diseases. On the basis of genetic predisposition, complex human diseases can be induced via the acquired environment factors such as personal age, diet, and physical activity (35).

In this case–control study, we confirmed that the TFBS polymorphism rs4134819 in *XAB2* gene was significantly associated with an increased GDM risk in all subjects and most subgroups (SBP > 110.03 mmHg, DBP > 69.44 mmHg, 2hPG > 7.10 mmol/L, HbA1c > 5.20%. and TG > 2.53 mmol/L) in the Guilin population. Furthermore, we have detected that age, glucose, and lipid metabolic indicators, including FPG, HbA1c, 1hPG, 2hPG, and TG, are also risk factors for GDM. A nomogram model constructed with the *XAB2* rs4134819 and the above clinical indicators suggested a good predictive performance with a diagnostic AUC of 0.931. These findings support the important role of rs4134819 in the pathogenesis of GDM.

Xeroderma Pigmentosum group A-binding protein 2 (XAB2) is a multifunctional protein playing a vital role in cellular processes such as transcription, splicing, DNA repair, and messenger RNA export (36). It was reported that XAB2 may exert as a regulator in hyperglycemia with chronic insulin (37, 38). We observed that *XAB2* rs4134819 was correlated with an elevated GDM risk in this study, and the FPRP analysis was performed to confirm the positive association. As predicted by bioinformatic tools, rs4134819 is located in the TFBS region of the *XAB2* gene. We further analyzed the potential biological functions of rs4134819 and found that rs4134819 C>T can alter the transcription factors binding to the promoter and act as an eQTL regulating gene transcription. Based on the above findings, it is speculated that this may be one of the biological mechanisms in which *XAB2* gene rs4134819 alters individual susceptibility to GDM.

Early pregnancy is a critical period before the onset of GDM and also a critical stage for fetal growth and development. It provides a unique opportunity for the prevention and treatment of uncertain maternal and child diseases in the future. Research supports the circulating biomarkers of the first trimester, such as blood sugar, fasting insulin, adiponectin, HDL-c, triglycerides, and C-reactive protein (39), which may predict an enhanced possibility of GDM risk. The deeper link of indicators of glycolipid metabolism to GDM may be that the glucose homeostasis is affected by disturbed lipid metabolism (40), while high triglycerides and elevated free fatty acids (FFAs) can generate oxidative stress and activate protein kinase C, coupled with the complex combined actions of a series of inflammatory factors, leading to insulin resistance and, ultimately, the development of hyperglycemia (41–43).

The nomogram model based on various risk variables has been regarded as a useful tool for GDM risk prediction in recent years. Wu et al. performed a nomogram of GDM based on maternal age, pre-pregnancy BMI, and OGTT, and obtained a diagnostic AUC of 0.872 (44). There were also several studies constructing GDM predictive models according to general conditions and laboratory indicators, such as TG, HDL-c, being overweight or obese before pregnancy, a family history of diabetes, a history of GDM, and a sedentary lifestyle (45–47). Although these models′ predictive powers were acceptable, lack of precise genetic indicators may decrease the predictive effect of the model. Our study established the dynamic nomogram by combining the genetic variant rs4134819 and significant clinical indicators (age, FPG, HbA1c, 1hPG, 2hPG, and TG), which demonstrated a good performance with an AUC of 0.931, a sensitivity of 0.934, and a specificity of 0.793. This suggests that in the construction of risk prediction models for complex diseases, including GDM, it is necessary to consider key genetic factors.

Our study has some limitations. First, the small sample analyzed in our previous chip screening stage may reduce statistical efficiency and cause the deviation of finding clues. Second, the subjects of this case–control study were selected from the hospital; thus, the selection bias was inevitable. Third, the biological function of the genetic variant was only predicted by bioinformatic tools and was not validated by molecular experiments. Fourth, the risk factors used to construct the nomogram were limited and we did not adopt the multi-center validation for the model’s predictive power. It is hoped that future studies will comprehensively consider multiple factors in establishing nomogram model and validate its predictive effect in different regions and population.

In conclusion, this study supports the significant association between *XAB2* rs4134819 C>T and the pathogenesis of GDM. Regulating the binding efficiency of transcription factors (CPE bind and GATA-1) and promoters and affecting the transcription of functional genes may be a potential mechanism. The dynamic nomogram constructed by genetic and clinical risk factors can effectively identify pregnant women with high GDM risk in early pregnancy.

## Data Availability

The data presented in the study are deposited in Dryad repository, accession number https://doi.org/10.5061/dryad.rv15dv4j7.
