# Supplementary material for: Association and function analysis of genetic variants and the risk of gestational diabetes mellitus in a southern Chinese population
Source: Front Endocrinol (Lausanne). 2024 Dec 24;15:1476222. doi: 10.3389/fendo.2024.1476222 (PMC11703716; doi:10.3389/fendo.2024.1476222)
Supplement: Supplementary file 1 [file Table1.docx]

**Supplemental Table S1 Basic characteristic of selected functional polymorphism**

| **rs#** | **Position** | **Gene**：**Consequence** | **Wild-type allele** | **Odds ratio** | ***P*-value** | **Potential functional prediction** |
| --- | --- | --- | --- | --- | --- | --- |
|  |  |  |  |  |  |  |
| rs4134819 | chr19:7628345 | *XAB2* : Intron Variant *PET100* : 2KB Upstream Variant | C | 2.81 | 1.58*10-4 | TFBS |
| rs720918 | chr3:149125697 | *HLTF*\|\|*HPS3* | A | 0.31 | 4.91*10-4 | TFBS |
| rs2034410 | chr11:6890714 | *OR10A4*\|\|*OR2D2* | T | 0.30 | 2.66*10-4 | miRNA |
| rs11109509 | chr12:98517378 | *TMPO* : Intron Variant *TMPO-AS1* : 2KB Upstream Variant | A | 0.31 | 8.03*10-5 | TFBS |
| rs12524768 | chr6:33792701 | *LOC105375024* : Non Coding Transcript Variant | G | 0.24 | 6.04*10^-4^ | TFBS |
| Note:#, variant variables; TFBS, transcription factor binding site; miRNA, microRNA | | | | | | |

**Supplemental Table S2 Primers sequences of functional polymorphisms used for polymerase chain reaction (PCR)**

| **SNP** | **Forward Primers Sequence** | **Reverse Primers Sequence** |
| --- | --- | --- |
| rs4134819 | 5'-ACGTTGGATGGGGATCTAGGTAAAAGTCCG-3' | 5'-ACGTTGGATGAGGGAATGGGAAGAAGAGAG-3' |
| rs720918 | 5'-ACGTTGGATGAAAAGTGTGTGGCACCTCTC-3' | 5'-ACGTTGGATGTCATTATAGAAGGTGAAGGG-3' |
| rs2034410 | 5'-ACGTTGGATGGATGGATATGGTAATTACCC-3' | 5'-ACGTTGGATGGTTTGGGAATACAGTGTGATG-3' |
| rs11109509 | 5'-ACGTTGGATGGGTTTGCTTATGCCTGCCGA-3' | 5'-ACGTTGGATGCACTGTCTCTTACAAGGCAT-3' |
| rs12524768 | 5'-ACGTTGGATGCAGGCTTATCCAGCAACATC-3' | 5'-ACGTTGGATGACATTCCAGATGAACATGGG-3' |
